# Supplementary material for: The Receptor Tyrosine Kinase TrkA Is Increased and Targetable in HER2-Positive Breast Cancer
Source: Biomolecules. 2020 Sep 17;10(9):1329. doi: 10.3390/biom10091329 (PMC7564775; doi:10.3390/biom10091329)
Supplement: Supplementary file 1 [file biomolecules-10-01329-s001.pdf]

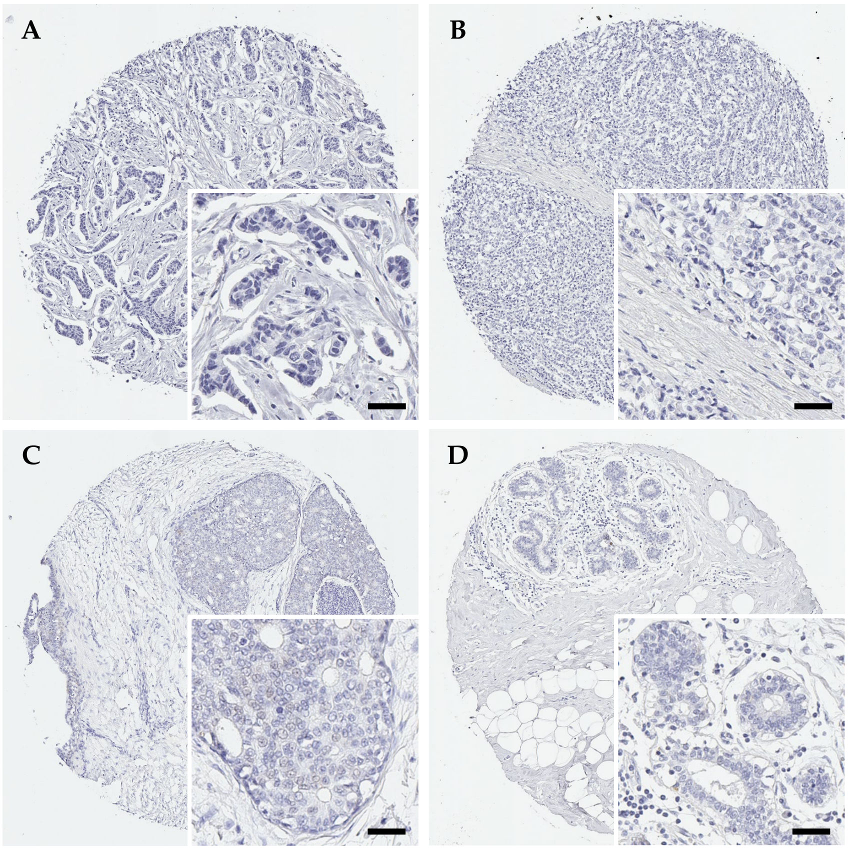

Supplementary Figure 1. IgG isotype controls staining for the immunohistochemical detection of TrkA in human breast cancers. The specificity of the TrkA antibody used for immunohistochemistry was validated through the use of an IgG isotype control in a matched case of (A) invasive ductal carcinoma (IDC), (B) invasive lobular carcinoma (ILC), © ductal carcinoma in situ (DCIS) and (D) normal breast tissue. Representative images of IgG isotype control immunolabelling are shown. Scale bars: 50 µm. Original magnification: x50 and x400 for entire cores and higher magnification regions, respectively.
